# Atherosclerotic spontaneous coronary artery dissection (A-SCAD) in a patient with COVID-19: case report and possible mechanisms

**DOI:** 10.1093/ehjcr/ytaa133

**Published:** 2020-05-12

**Authors:** Remo Albiero, Giuseppe Seresini

**Affiliations:** Interventional Cardiology Unit, Cardiovascular Department, Sondrio Hospital, Sondrio (SO), Italy

**Keywords:** Coronary artery dissection, SCAD, A-SCAD, COVID-19, PCI, ACS, Case report

## Abstract

**Background:**

Spontaneous coronary artery dissection (SCAD) may be atherosclerotic (A-SCAD) or non-atherosclerotic (NA-SCAD) in origin. Contemporary usage of the term ‘SCAD’ is typically synonymous with NA-SCAD. COVID-19 could induce a vascular inflammation localized in the coronary adventitia and periadventitial fat and contribute to the development of an A-SCAD of a vulnerable plaque in a susceptible patient.

**Case summary:**

In this report we describe a case of a COVID-19 patient with past cardiac history of CAD who was admitted for acute coronary syndrome (ACS). Coronary angiography demonstrated the culprit lesion in the proximal LAD that presented with a very complex and unusual morphology, indicative of an A-SCAD. The diagnosis of A-SCAD was supported by the presence of a mild stenosis in the same coronary segment in the last angiogram performed 3 years previously. He was successfully treated by PCI, had a favourable course of the COVID-19 with no symptoms of pneumonia, and was discharged from the hospital after two negative tests for SARS-CoV-2.

**Discussion:**

A higher index of suspicion of A-SCAD is needed in patients with suspected or confirmed COVID-19 presenting with ACS. The proposed approach with ‘thrombolysis first’ for treating STEMI patients with suspected or confirmed COVID-19 infection could be unsafe in the case of underlying A-SCAD.

**Learning points**

- A high index of suspicion of A-SCAD is needed in patients presenting with ACS during the COVID-19 pandemic.
- Use of thrombolysis in a suspected or confirmed COVID-19 case presenting with STEMI might be unsafe in the case of an underlying A-SCAD.

## Introduction

A significant association has been reported between respiratory infections, especially influenza, and acute myocardial infarction (AMI).^1^ Coronavirus disease 2019 (COVID-19) has rapidly grown into a pandemic, and a large proportion of patients have underlying cardiovascular disease and/or cardiac risk factors.^2^ COVID-19 is associated with a high inflammatory burden that can induce vascular inflammation, myocarditis, and cardiac arrhythmias.^2^ Spontaneous coronary artery dissection (SCAD) is defined as a non-traumatic and non-iatrogenic separation of the coronary arterial wall. Pressure-driven expansion of the false lumen by an enlarging intramural haematoma (IMH) may lead to luminal encroachment and subsequent myocardial ischaemia and infarction.^3^ SCAD may be atherosclerotic (A-SCAD)^4^ or non-atherosclerotic (NA-SCAD)^3^ in origin. Contemporary usage of the term ‘SCAD’ is typically synonymous with NA-SCAD that can result in extensive dissection lengths, especially in the presence of arterial fragility from predisposing arteriopathies.^3^ A-SCAD, on the other hand, is a mechanistically distinct variant of SCAD and is typically limited in extent by medial atrophy and scarring.^5^ In this report, we describe the case of an A-SCAD in a COVID-19 patient with acute coronary syndrome (ACS).

## Timeline

**Table:** 

Day 1	Presentation to the Emergency Department with acute coronary syndrome and NSTEMI.
	Presence of ECG ST-T abnormalities in the precordial lead and ecocardiographic left ventricular wall motion abnormalities
Day 1	Coronary angiography demonstated an LAD complex culprit lesion that was identified angiographically as a spontaneous coronary dissection (SCAD).
	PCI was performed with a good final result and no procedural complications
Day 2	The patient had fever with suspicion of COVID-19. A swab test was performed to confirm the diagnosis.
Day 3	Real-time PCR confirmation of COVID-19 infection
Day 3	The patient was transferred in a dedicated COVID-19 hospital to continue the isolation
Day 3–9	Pharmacological treatment for COVID-19 was only supportive without use of antivirals. The patients had a favourable course of CIVID-19 with no symptoms of pneumonia
Day 12	Patient discharged from the dedicated COVID-19 hospital after two negative tests for SARS-CoV-2 performed at 24-h intervals.

## Case presentation

### History and examination findings

A 70-year-old man was admitted to the Emergency Department in March 2020 with persistent severe chest pain (grade 8/10) which started 3 h before admission. The cardiovascular and respiratory examinations on admission were normal. He had an elevated blood pressure of 155/100 mmHg, a regular heart rate of 63 b.p.m., an oxygen saturation rate of 90% on room air, and no respiratory compromise or fever. The cardiovascular risk factors were: smoking, hypertension, and type 2 diabetes. His medical therapy on admission was: aspirin 100 mg/day, ramipril 1.25 mg/day, bisoprolol 1.25 mg/day, and metformin 850 mg t.i.d. From his past cardiac history, it emerged that he underwent prior percutaneous coronary intervention (PCI) with implantation of a drug-eluting stent (DES) on the mid left circumflex artery (LCx) in 2007 and proximal LCx-OM1 in 2017. His initial high sensitivity troponin I was 84.7 ng/L (normal range <34).

### Laboratory tests

**Figure 1:**
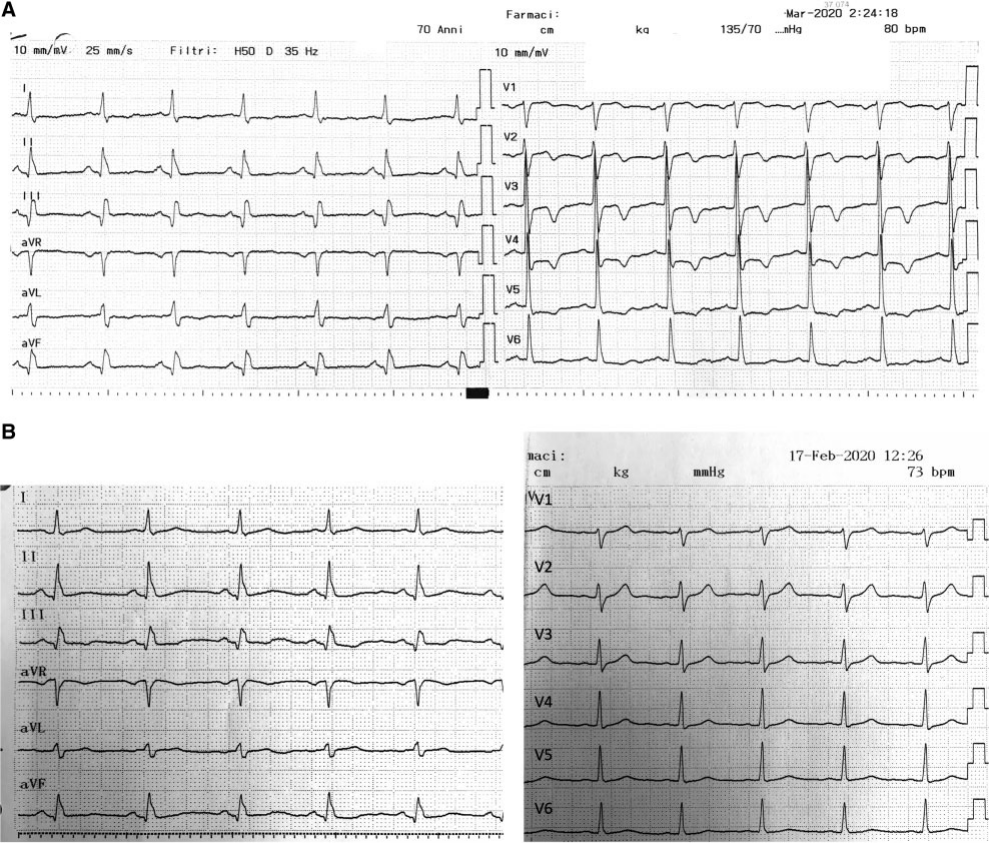
(*A*) Admission ECG, performed in March 2020, demonstrates ST-T abnormalities in the precordial leads. (B) ECG performed in February 2020 demonstrates absence of ST-T abnormalities in the precordial leads.

Blood count and inflammatory markers were in the normal range. Coagulation test demonstrated only an increased D-dimer of 736 ng/mL (normal range 0–710). The ECG (*Figure 1a*) demonstrated new ST-T abnormalities in the precordial leads not present in the last ECG performed 1 month before (*Figure 1b*). The echocardiogram demonstrated a left ventricular ejection fraction of 40–45% with akinesia in the LCx territory (described since 2008) and a severe hypokinesis in the left anterior ascending (LAD) territory.

### Hospital treatment

**Figure 2:**
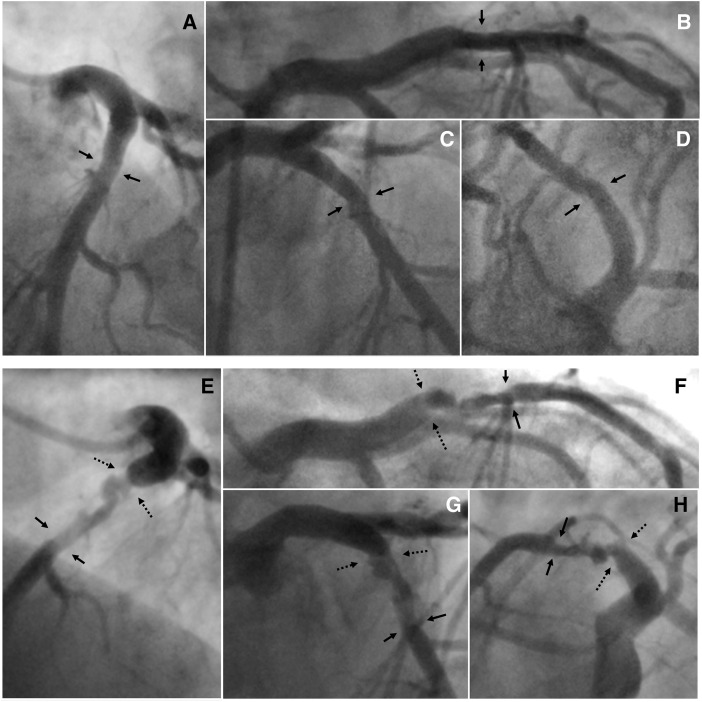
(*a*) Selective left coronary arteriograms performed in 2017: (A) in LAO cranial view; (B) in RAO caudal view; (C) in RAO cranial view; (D) in LAO caudal view. The black arrows indicate the presence of a mild stenosis in the proximal segment of the left anterior descending (LAD) coronary artery. (*b*) Selective left coronary arteriograms performed in March 2020: (E) in LAO cranial view; (F) in RAO caudal view; (G) in RAO cranial view; (H) in LAO caudal view. The dotted black arrows indicate where the A-SCAD started, while the solid black arrow shows where the A-SCAD ended that was located exactly at the origin of the first two encountered branches (the first septal and the first diagonal branch).

**Figure 3:**
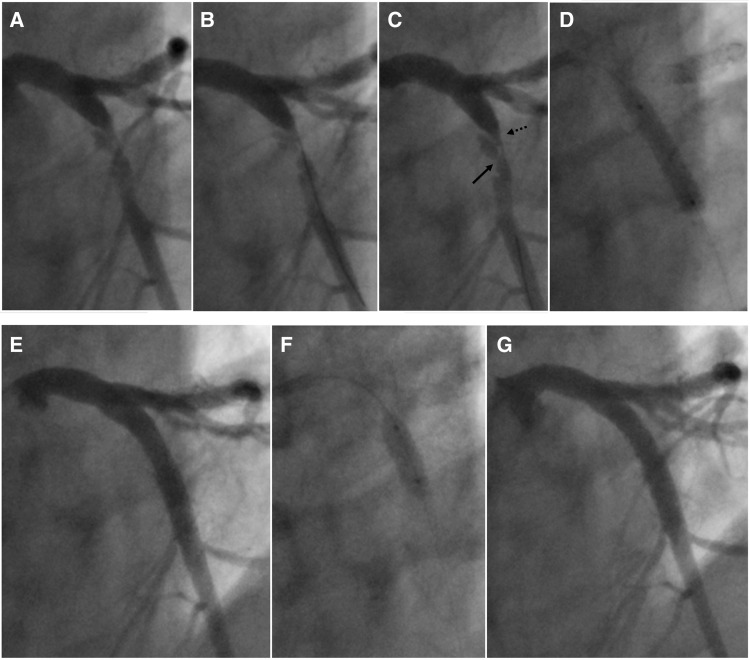
(*a*) PCI of the proximal LAD performed in RAO cranial view projection. (A) Before guidewire crossing; (B) after the workhorse guidewire had easily crossed the lesion; (C) the solid black arrow indicates the site of the intimal tear which is distal to the entry point (dotted arrow); (D) direct drug-eluting stent (DES) implantation (4.0 × 23 mm at 18 atm). (*b*) (E) After stenting; (F) stent post-dilatation with an NC balloon 4.5 × 12 mm inflated at 22 atm; (G) Final good angiographic result.

The patient was treated with i.v. heparin, sublingual nitroglycerin, and clopidogrel. The coronary angiogram, performed 10 h after admission, showed a moderate in-stent restenosis on LCx-OM and a moderate right coronary artery (RCA) stenosis. The culprit lesion was the proximal LAD that presented with a very complex and unusual plaque morphology, indicative of a coronary artery dissection: an abrupt change in arterial calibre both at the beginning and at the end (exactly at the origin of the first septal and diagonal branches), two lumina separated by a radiolucent flap evident in the proximal and mid portion of the dissection, a spiral pattern, no endoluminal thrombus, TIMI 3 flow, and a very small mid-distal intimal tear (*Figure 2b*, Supplementary material online, *Video S1*). The angiographic characteristics were also in accordance with the aforementioned definition of SCAD.^3^ In our case, the IMH probably propagated antegrade and retrograde, compressing the arterial lumen and causing ischaemia. In addition, the very small mid-distal intimal tear that we observed was probably the site of intramural egress of blood. The diagnosis of A-SCAD was furtherly supported by the presence of a mild stenosis in the same coronary segment in the last angiogram performed in 2017 (*Figure 2a*). The LAD lesion at the site of A-SCAD was then treated by PCI: easily crossed with a workhorse guidewire (*Figure 3a*, panel B and C) followed by direct implantation of a drug-eluting stent 4.0 × 23 mm (*Figure 3a*, panel D), that was post-dilated with an NC balloon 4.5 × 12 mm inflated at 22 atm (*Figure 3b*, panel F), with and excellent angiographic final result (*Figure 3b*, Panel G) and final TIMI 3 flow. The day after the procedure, the patient developed fever (temperature >38°C) which prompted the suspicion of COVID-19. The patient was immediately put in isolation in a dedicated room. The diagnosis of COVID-19 was confirmed by real-time PCR test and the patient was transferred in a dedicated COVID-19 hospital to continue the isolation and the pharmacological treatment for COVID-19 that was only supportive without use of antivirals.

### Clinical course and laboratory test outcomes

The patient had a favourable clinical course of COVID-19 with no symptoms of pneumonia. In addition, he had no further episodes of chest pain. The echocardiogram demonstrated the disappearance of the left ventricular severe hypokinesis in the LAD territory present at admission that was associated with normalization of the electrocardiographic ST-T abnormalities in the precordial leads. He was finally discharged 12 days later, after two negative tests for SARS-CoV-2 at 24 h intervals, with the following medical therapy: aspirin 100 mg/day, clopidogrel 75 mg/day, pantoprazole 40 mg/day, atorvastatin 40 mg/day, bisoprolol 1.25 mg/day, and metformin 850 mg t.i.d.

## Discussion

A large proportion of patients with COVID-19 have an underlying cardiovascular disease.^2^ In this setting, there is a limited amount of data supporting the hypothesis that the inflammatory response resulting from COVID-19 may place susceptible patients at risk of A-SCAD. Herein, we report the second case of an A-SCAD in a patient with COVID-19. However, unlike the first case reported,^6^ in our case the angiographic diagnosis of coronary artery dissection was not confirmed by intracoronary imaging (considered the gold standard for diagnosis of NA-SCAD, to visualize the presence of an IMH and its compression of the true lumen) because there was a clear identification of the IMH egression site and an evident compression of the true lumen.

### Relationship between acute respiratory viral infections and ACS

For decades, increased morbidity and mortality related to ACS and other cardiac conditions has been recognized during influenza epidemics. A study of 34 000 autopsies^7^ showed that MI is 30% more likely to happen during influenza season compared with off-season weeks. We can therefore infer that acute respiratory viral infections, more likely than not, have a role in triggering the development of a coronary event.^8^ Acute viral infections might also have direct inflammatory effects on atherosclerotic plaques and coronary arteries.^8^ When compared with patients with stable CAD, patients with ACS have higher inflammatory activity and the culprit lesion has more infiltrating inflammatory cells (i.e. macrophages, T cells, and neutrophils) than other lesions in the coronary tree.^9^ People dying of acute systemic infections have a substantially higher number of macrophages and T cells in the coronary adventitia and periadventitial fat, and more dendritic cells in the intima and media than people who died without infection.^10^ In addition, coronary vulnerable plaques represent a separate immunological compartment from blood with lymphocytes characterized by a high level of T-cell activation.^11^ The presence of these cells in the coronary adventitia and periadventitial fat, that in our case were possibly activated further by the SARS-CoV-2 viral infection, might have contributed to the development of the A-SCAD by the production of cytokines and protease that could have destabilized a vulnerable plaque.^8^ In addition, the aforementioned advential inflammation could facilitate the primary disruption of a vasa vasorum micro-vessel leading to haemorrhage directly into the tunica media that has been proposed by the ESC/ACCA position paper on spontaneous coronary artery dissection^12^ as a causal event of the ‘outside-in’ mechanism of SCAD. This mechanism is supported by the increasing evidence indicating the importance of adventitial inflammation in atherosclerotic vulnerable plaques. Human atherosclerotic coronary plaques with large lipid cores have a significantly greater number of macrophages in their periadventitial fat than do fibrocalcific and non-atherosclerotic arterial segments, which suggests the involvement of adventitial inflammatory cells in plaque vulnerability and that the adventitia and periadventitial fat may function as a unit.^13^

### Risk of thrombolysis in SCAD

The approach with ‘thrombolysis first’ for the treatment of STEMI patients with COVID-19, as recently recommended for Chinese medical institutions and promoted worldwide for peer reference,^14^ could be unsafe in the case of STEMI and spontaneous coronary dissection, worsening the condition of patients as reported by Shamloo *et al*.^15^ Therefore, we suggest the revision of the recent Chinese recommendations in addition to the ACC/SCAI statement proposing that ‘fibrinolysis can be considered an option for the relatively stable STEMI patient with active COVID-19’, after careful consideration of possible patient benefit vs. the risks of cath-lab personnel exposure to the virus.

## Conclusions

COVID-19, which has rapidly grown into a pandemic, is associated with a high inflammatory burden that could induce a vascular inflammation localized in the coronary adventitia and periadventitial fat and contribute to the development of an A-SCAD of a vulnerable plaque in a susceptible patient. A higher index of suspicion of A-SCAD is needed in patients with suspected or confirmed COVID-19 presenting with ACS. The proposed approach with ‘thrombolysis first’ for treating STEMI patients with suspected or confirmed COVID-19 infection could be unsafe in the case of underlying A-SCAD.

## Lead author biography

**Figure:**
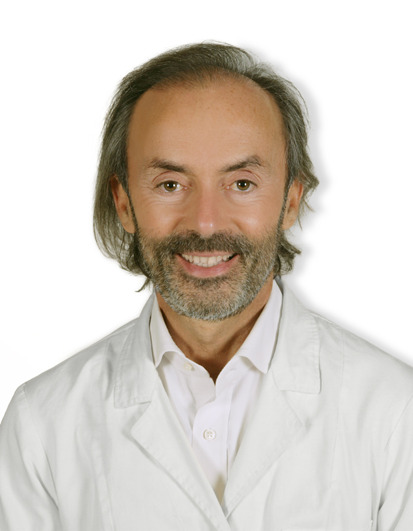


Remo Albiero, MD, graduated in Medicine in Italy in 1987. In 1991 he achieved the Specialization in Cardiology at the University of Verona. From 1997 to 2002 he worked at the Catheterization Laboratory, EMO Centro Cuore Columbus, Milan, Italy (Director Dr Antonio Colombo) as Senior Associate, and practised interventional cardiology. From 2002 to 2019, he was Director of the Cardiac Catheterization Laboratory in Ome, Brescia, Italy. From 2020 he has worked as an interventional cardiologist at Sondrio Hospital (Sondrio, Italy). He has particular expertise in the performance of complex coronary interventions. He is author of many academic manuscripts and book chapters, and belongs to the faculty of National and International Cardiology conferences.

## Supplementary material

Supplementary material is available at *European Heart Journal - Case Reports* online.

**Slide sets:** A fully edited slide set detailing this case and suitable for local presentation is available online as Supplementary data.

**Consent:** The authors confirm that written consent for submission and publication of this case report including images and associated text has been obtained from the patient in line with COPE guidance.

**Conflict of interest:** none declared.

## Supplementary Material

ytaa133_Supplementary_Data
