# Supplementary material for: Atherosclerotic spontaneous coronary artery dissection (A-SCAD) in a patient with COVID-19: case report and possible mechanisms
Source: Eur Heart J Case Rep. 2020 May 12;4(FI1):1–6. doi: 10.1093/ehjcr/ytaa133 (PMC7239234; doi:10.1093/ehjcr/ytaa133)
Supplement: ytaa133_Supplementary_Data [file ytaa133_supplementary_data.zip › ytaa133_Supplementary_Data/EHJ-CR-D-20-00531 Slide-Set.pptx]

## Slide 1
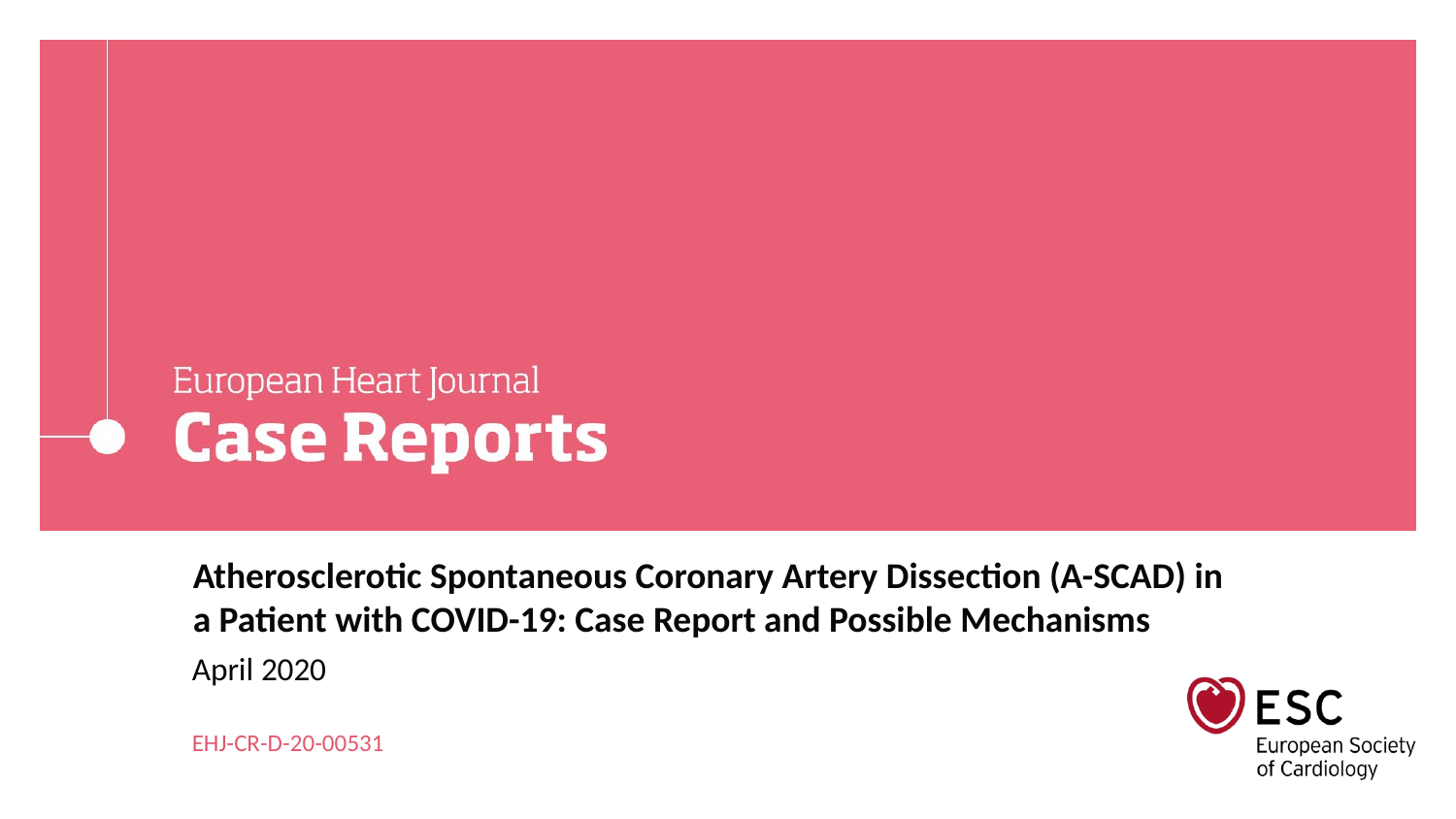

# Atherosclerotic Spontaneous Coronary Artery Dissection (A-SCAD) in a Patient with COVID-19: Case Report and Possible Mechanisms
April 2020
EHJ-CR-D-20-00531

## Slide 2
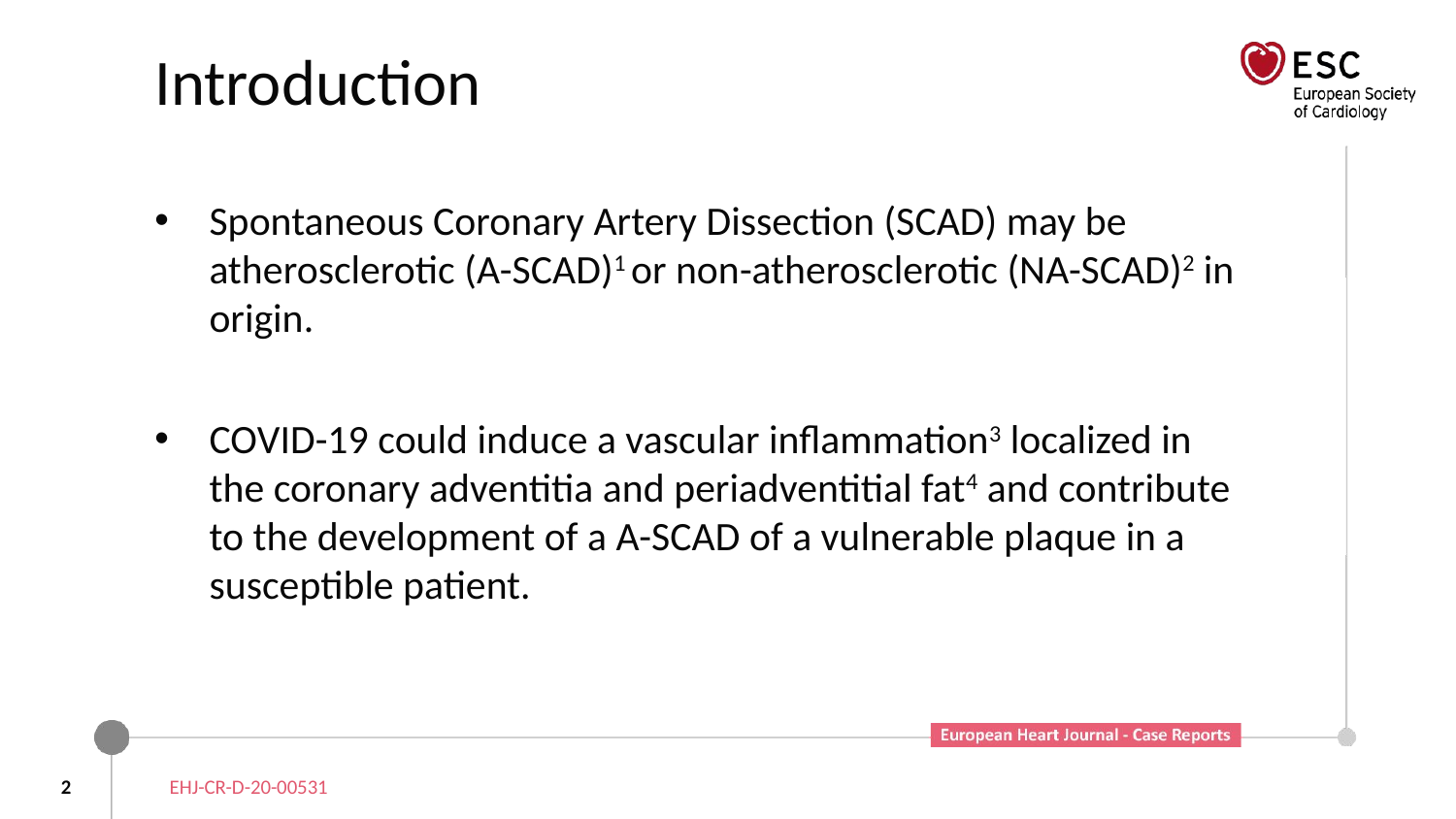

# Introduction
Spontaneous Coronary Artery Dissection (SCAD) may be atherosclerotic (A-SCAD)1 or non-atherosclerotic (NA-SCAD)2 in origin.
COVID-19 could induce a vascular inflammation3 localized in the coronary adventitia and periadventitial fat4 and contribute to the development of a A-SCAD of a vulnerable plaque in a susceptible patient.
2
EHJ-CR-D-20-00531

## Slide 3
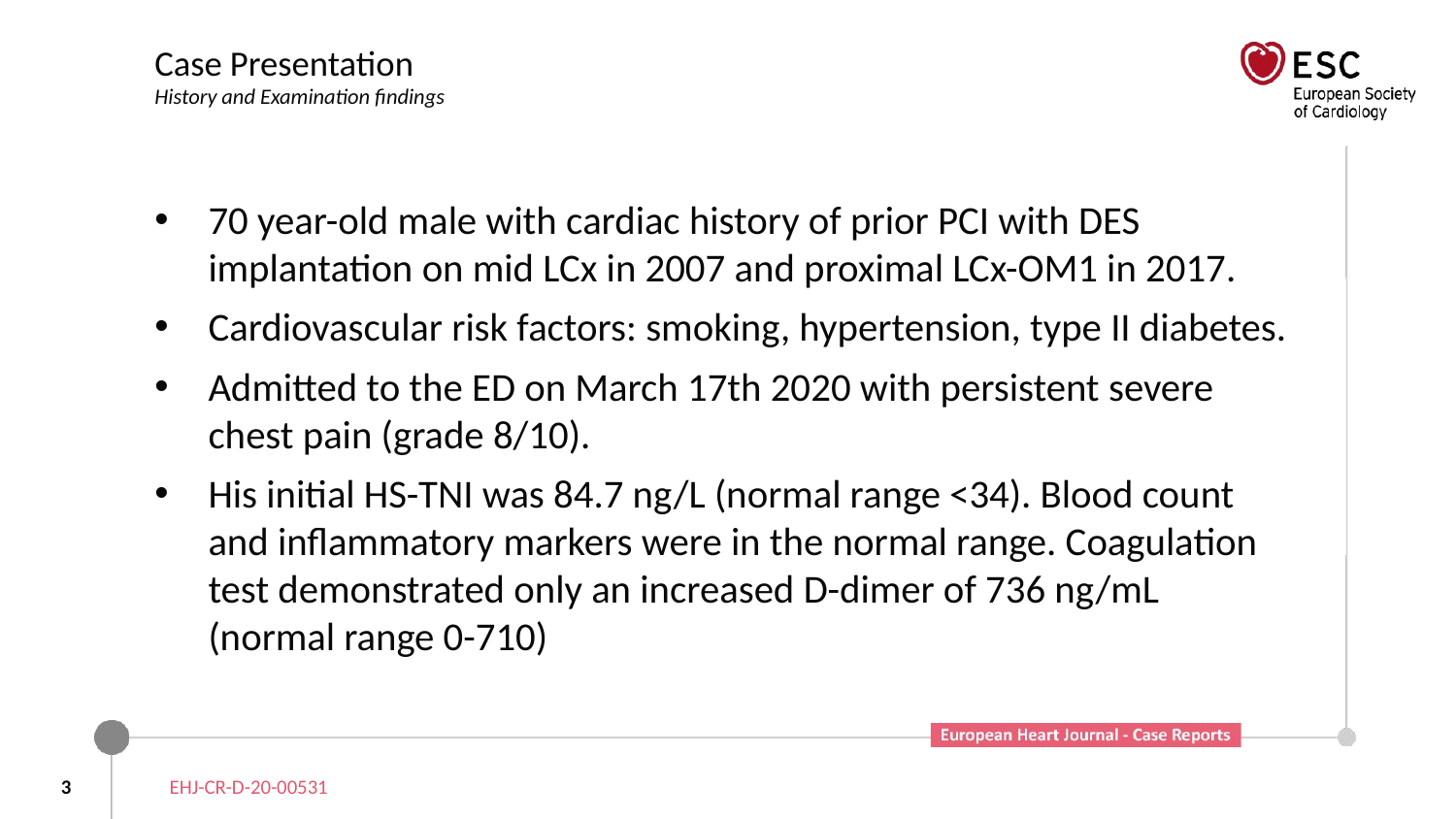

# Case Presentation History and Examination findings
70 year-old male with cardiac history of prior PCI with DES implantation on mid LCx in 2007 and proximal LCx-OM1 in 2017.
Cardiovascular risk factors: smoking, hypertension, type II diabetes.
Admitted to the ED on March 17th 2020 with persistent severe chest pain (grade 8/10).
His initial HS-TNI was 84.7 ng/L (normal range <34). Blood count and inflammatory markers were in the normal range. Coagulation test demonstrated only an increased D-dimer of 736 ng/mL (normal range 0-710)
3
EHJ-CR-D-20-00531

## Slide 4
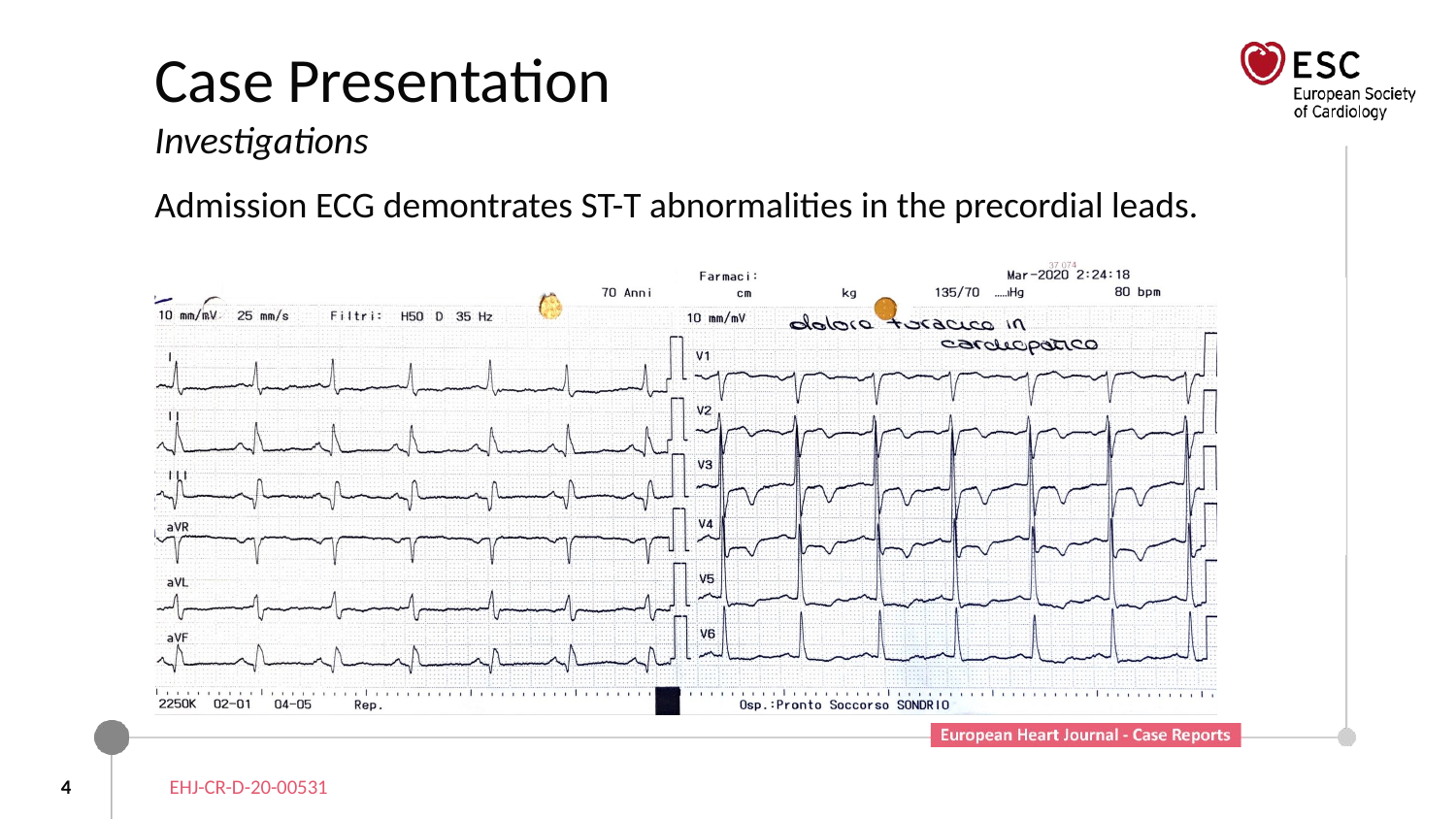

# Case Presentation Investigations
Admission ECG demontrates ST-T abnormalities in the precordial leads.
4
EHJ-CR-D-20-00531

## Slide 5
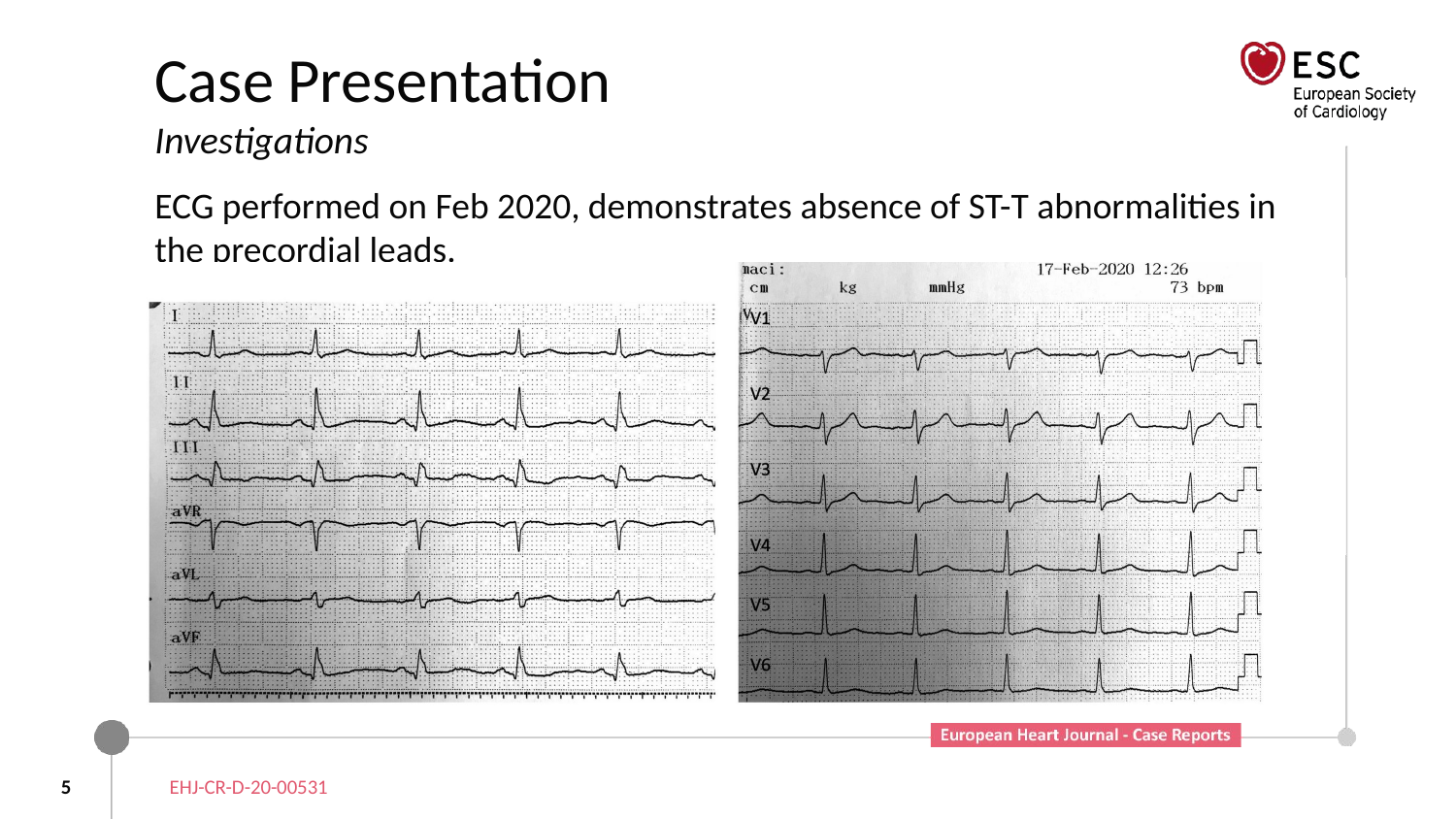

# Case Presentation Investigations
ECG performed on Feb 2020, demonstrates absence of ST-T abnormalities in the precordial leads.
5
EHJ-CR-D-20-00531

## Slide 6
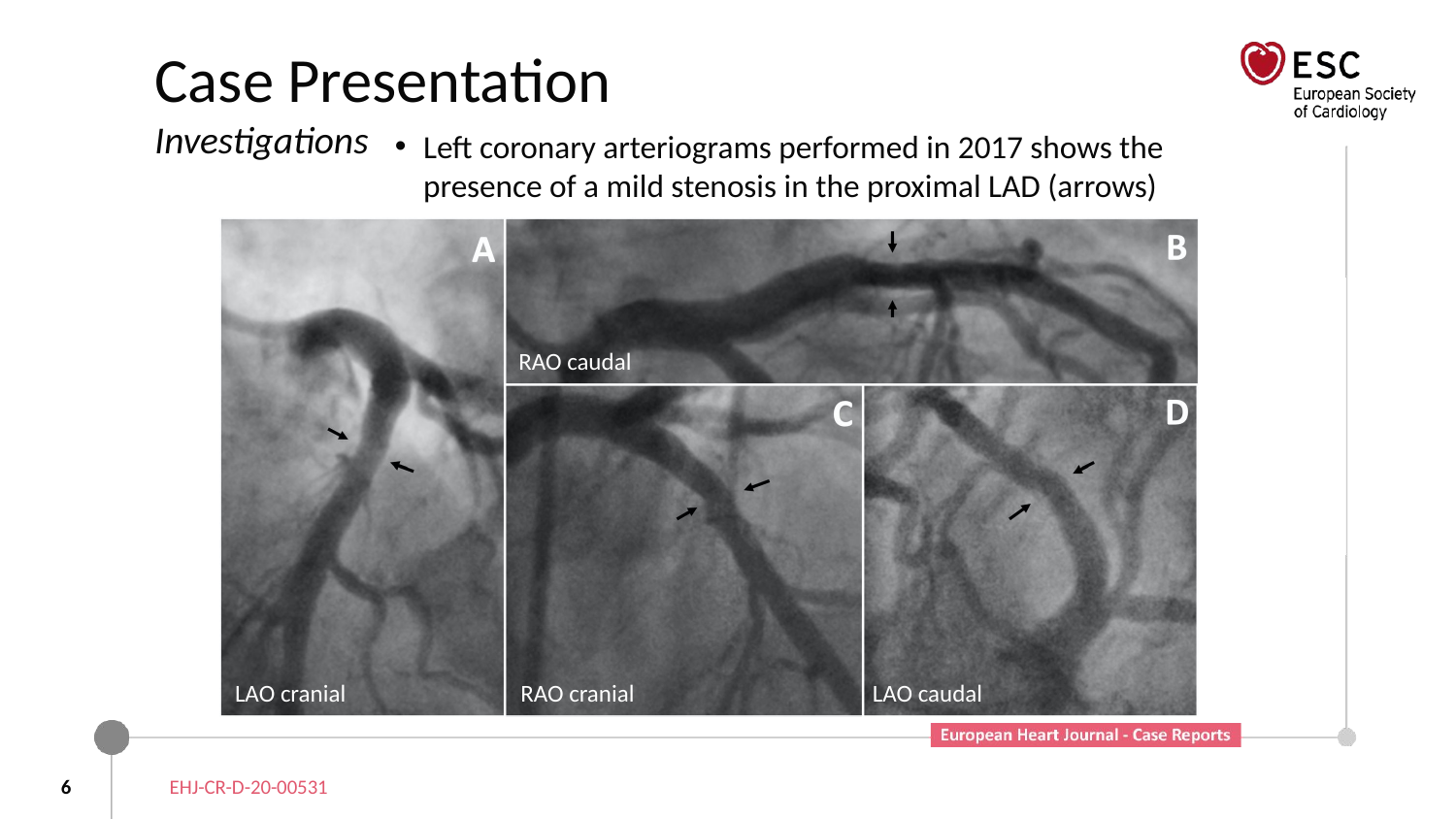

# Case Presentation Investigations
Left coronary arteriograms performed in 2017 shows the presence of a mild stenosis in the proximal LAD (arrows)
RAO caudal
LAO caudal
RAO cranial
LAO cranial
6
EHJ-CR-D-20-00531

## Slide 7
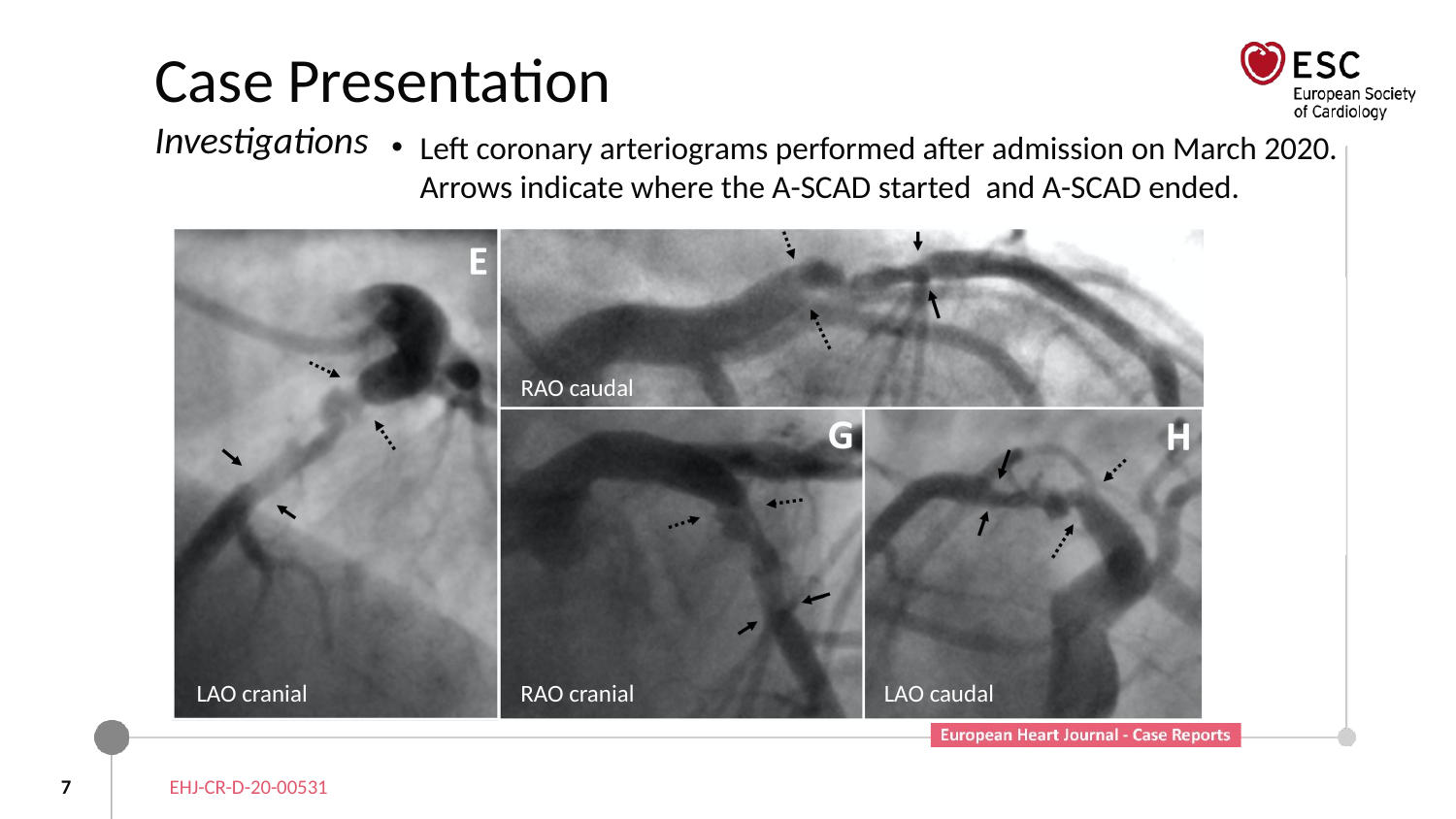

# Case Presentation Investigations
Left coronary arteriograms performed after admission on March 2020. Arrows indicate where the A-SCAD started and A-SCAD ended.
RAO caudal
LAO cranial
LAO caudal
RAO cranial
7
EHJ-CR-D-20-00531

## Slide 8
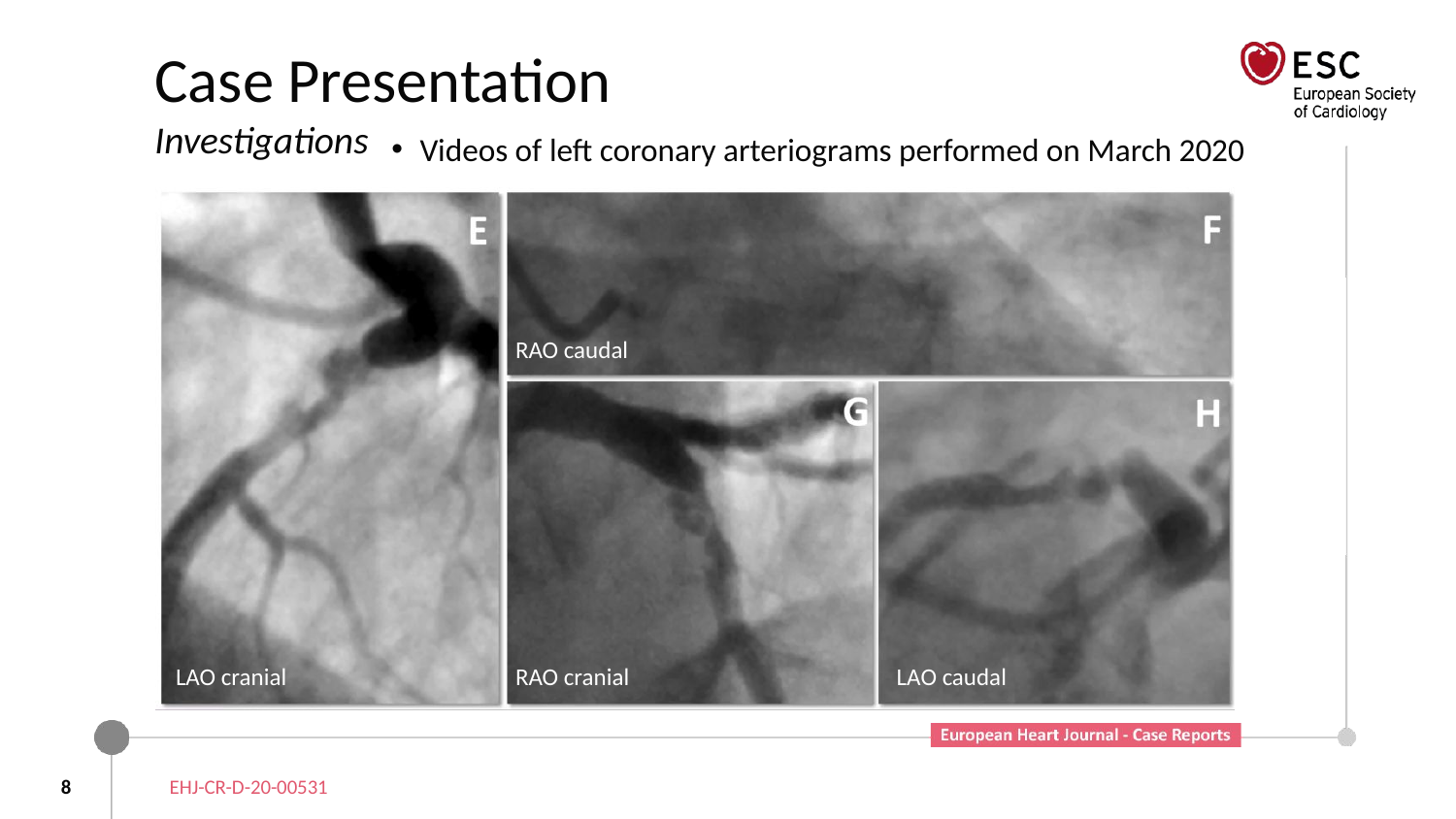

# Case Presentation Investigations
Videos of left coronary arteriograms performed on March 2020
RAO caudal
LAO cranial
LAO caudal
RAO cranial
8
EHJ-CR-D-20-00531

## Slide 9
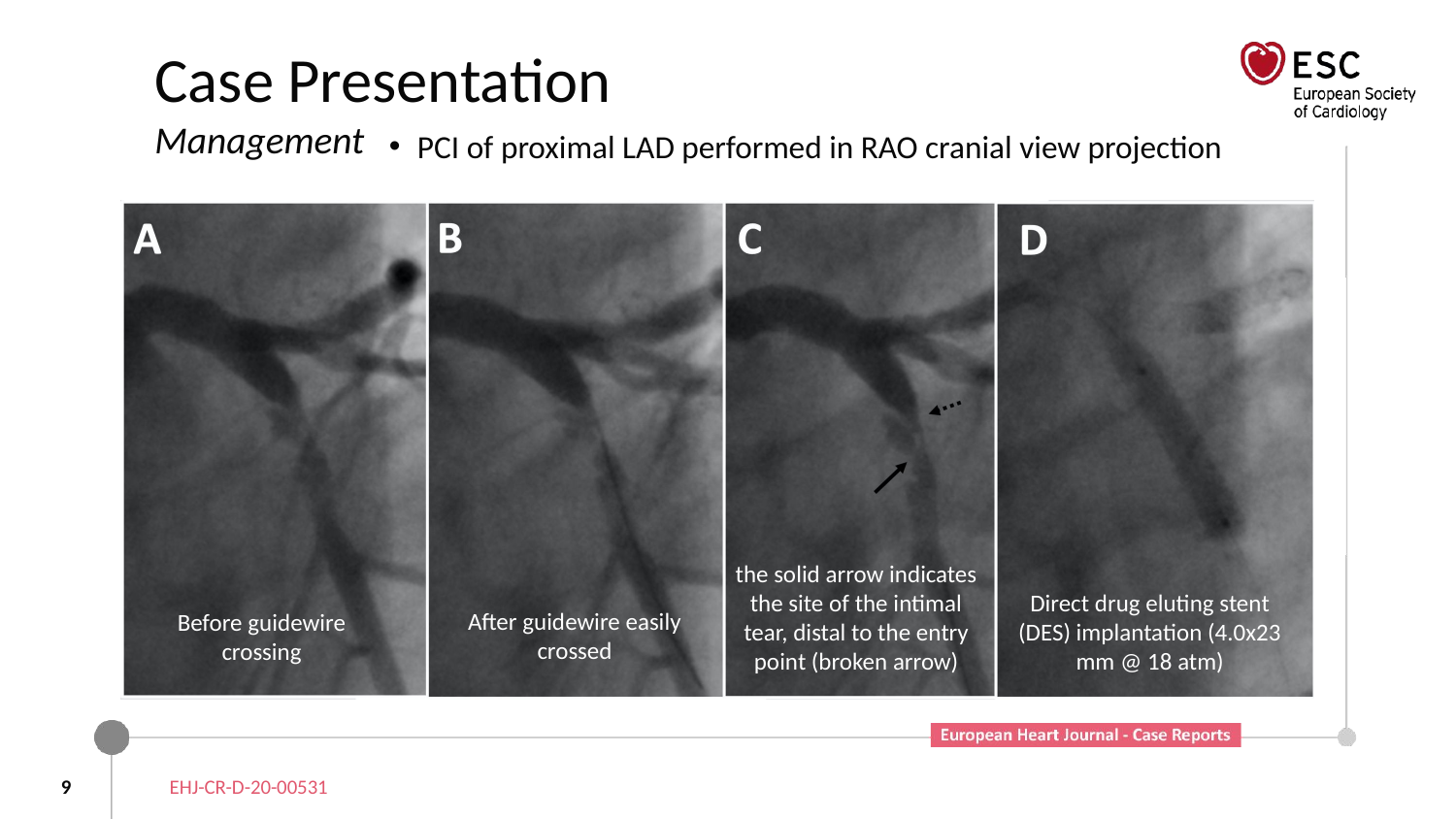

# Case Presentation Management
PCI of proximal LAD performed in RAO cranial view projection
the solid arrow indicates the site of the intimal tear, distal to the entry point (broken arrow)
Direct drug eluting stent (DES) implantation (4.0x23 mm @ 18 atm)
After guidewire easily crossed
Before guidewire crossing
9
EHJ-CR-D-20-00531

## Slide 10
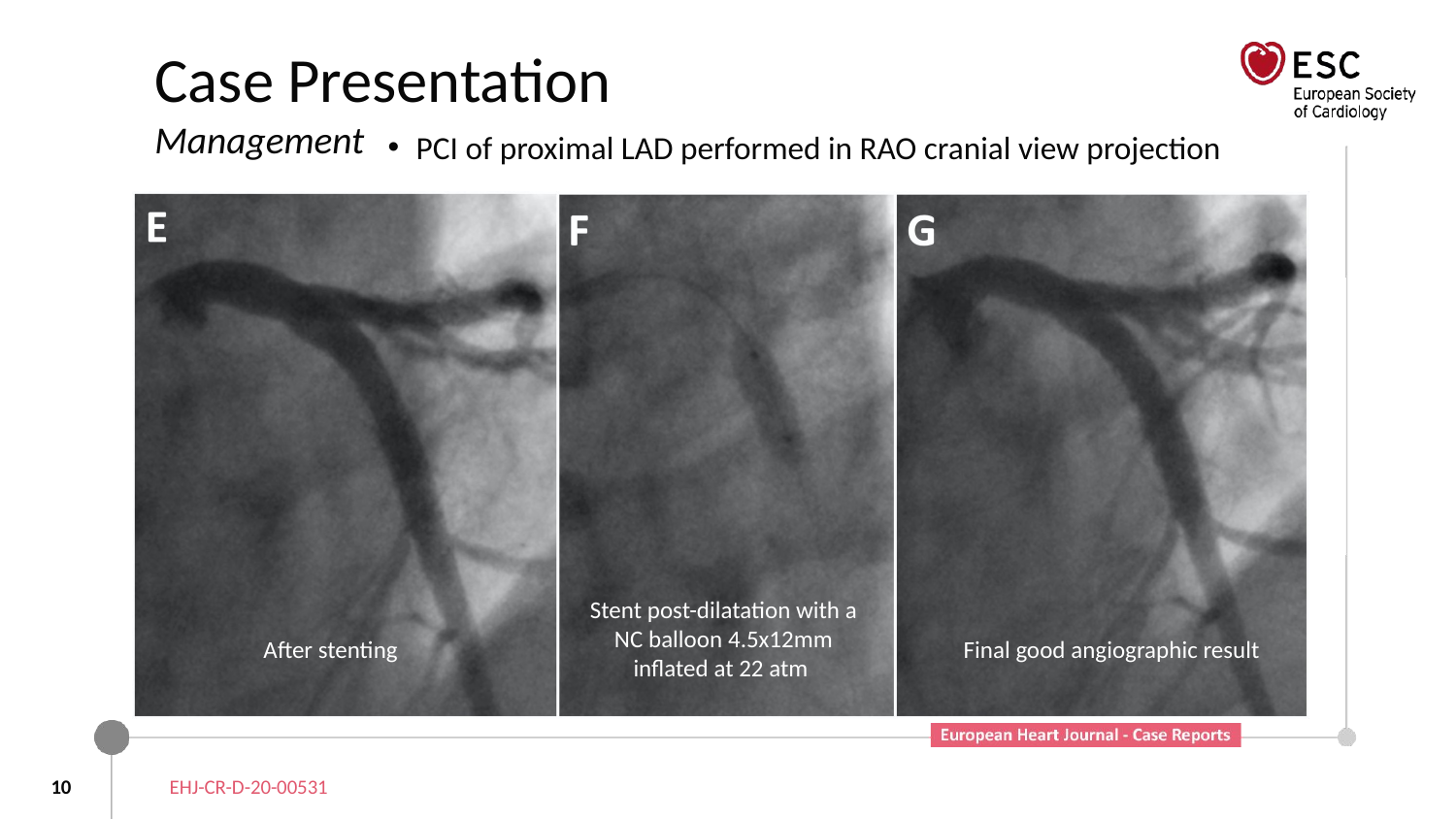

# Case Presentation Management
PCI of proximal LAD performed in RAO cranial view projection
Stent post-dilatation with a NC balloon 4.5x12mm inflated at 22 atm
Final good angiographic result
After stenting
10
EHJ-CR-D-20-00531

## Slide 11
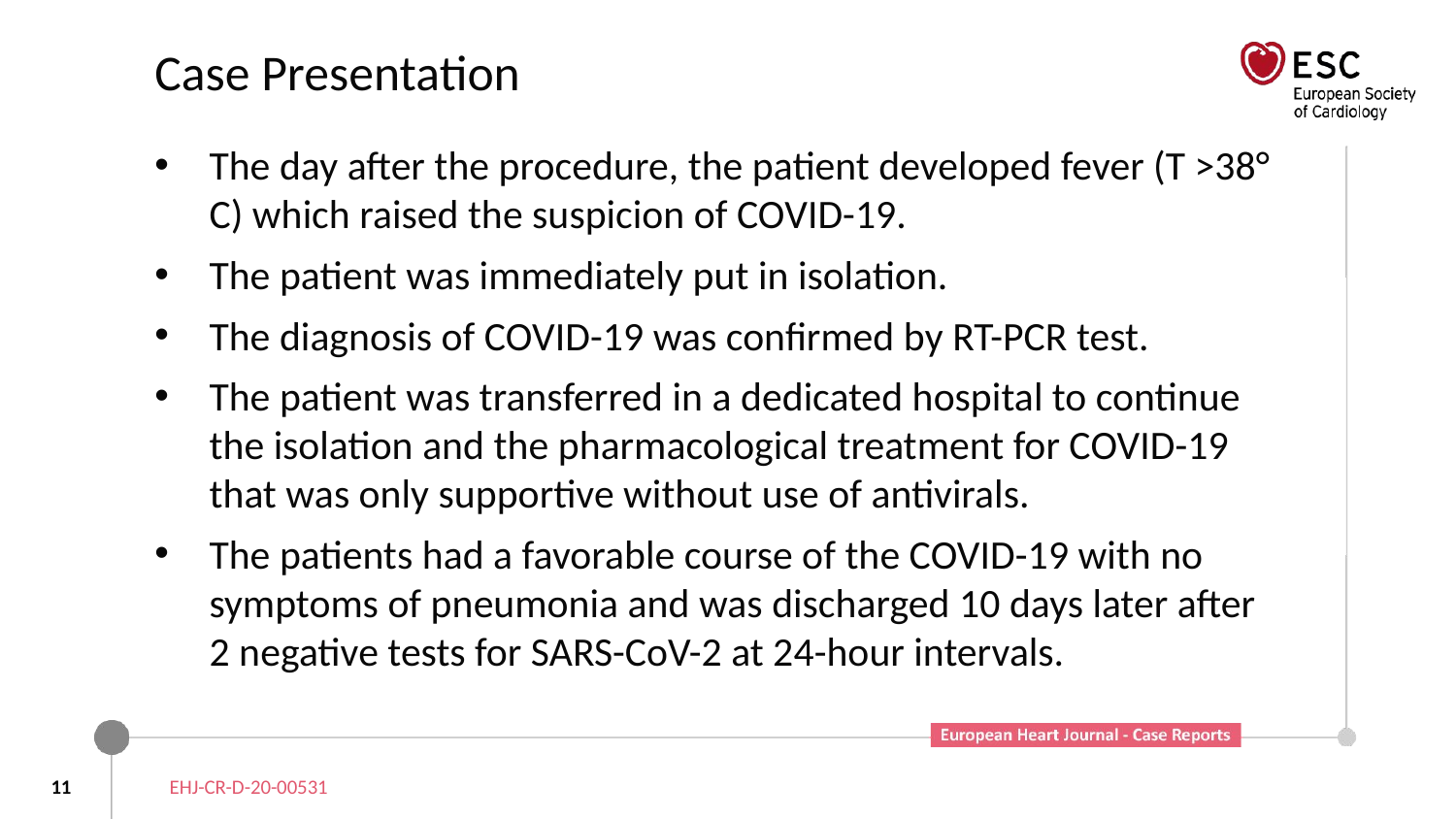

# Case Presentation
The day after the procedure, the patient developed fever (T >38° C) which raised the suspicion of COVID-19.
The patient was immediately put in isolation.
The diagnosis of COVID-19 was confirmed by RT-PCR test.
The patient was transferred in a dedicated hospital to continue the isolation and the pharmacological treatment for COVID-19 that was only supportive without use of antivirals.
The patients had a favorable course of the COVID-19 with no symptoms of pneumonia and was discharged 10 days later after 2 negative tests for SARS-CoV-2 at 24-hour intervals.
11
EHJ-CR-D-20-00531

## Slide 12
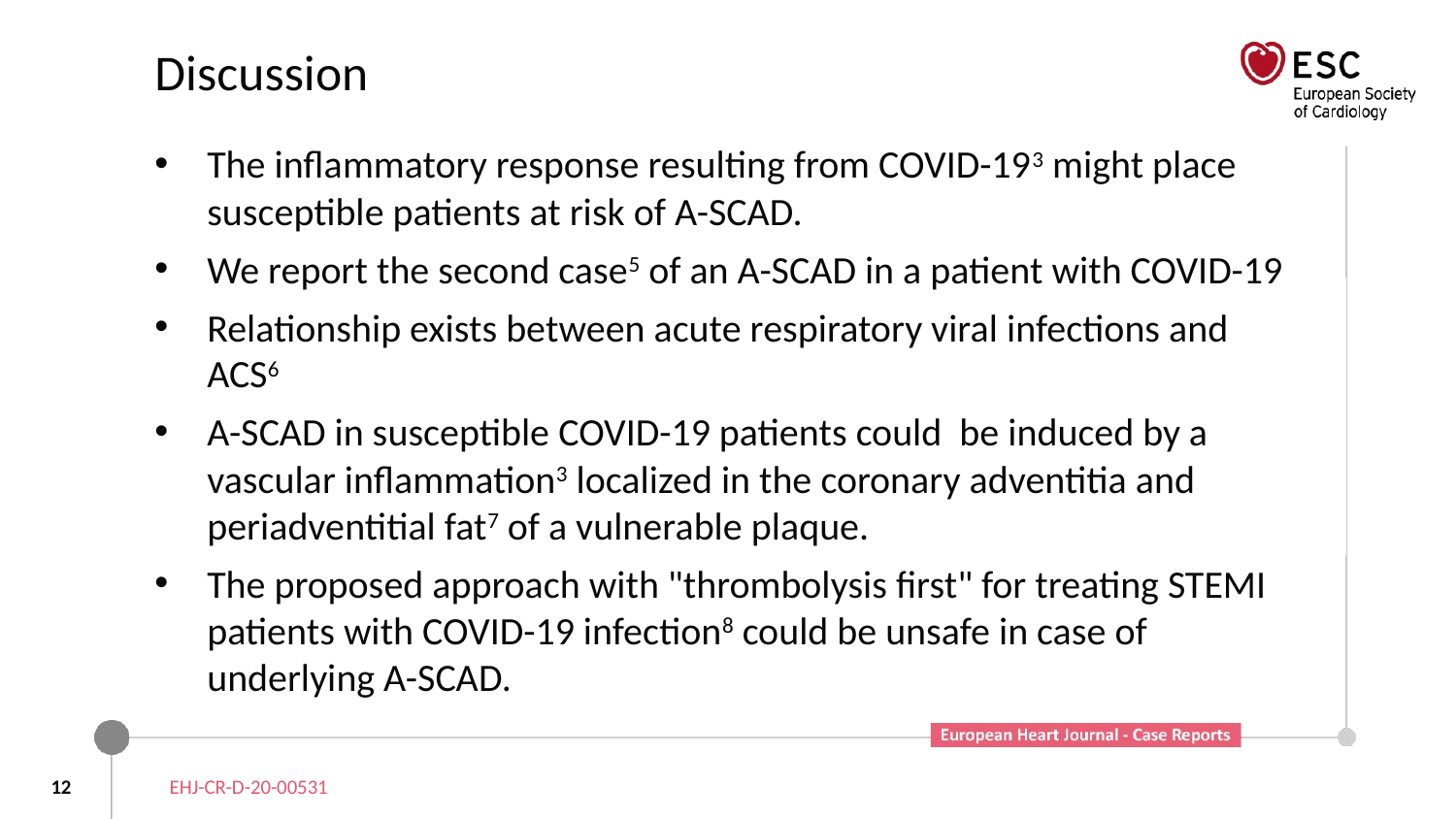

# Discussion
The inflammatory response resulting from COVID-193 might place susceptible patients at risk of A-SCAD.
We report the second case5 of an A-SCAD in a patient with COVID-19
Relationship exists between acute respiratory viral infections and ACS6
A-SCAD in susceptible COVID-19 patients could be induced by a vascular inflammation3 localized in the coronary adventitia and periadventitial fat7 of a vulnerable plaque.
The proposed approach with "thrombolysis first" for treating STEMI patients with COVID-19 infection8 could be unsafe in case of underlying A-SCAD.
12
EHJ-CR-D-20-00531

## Slide 13
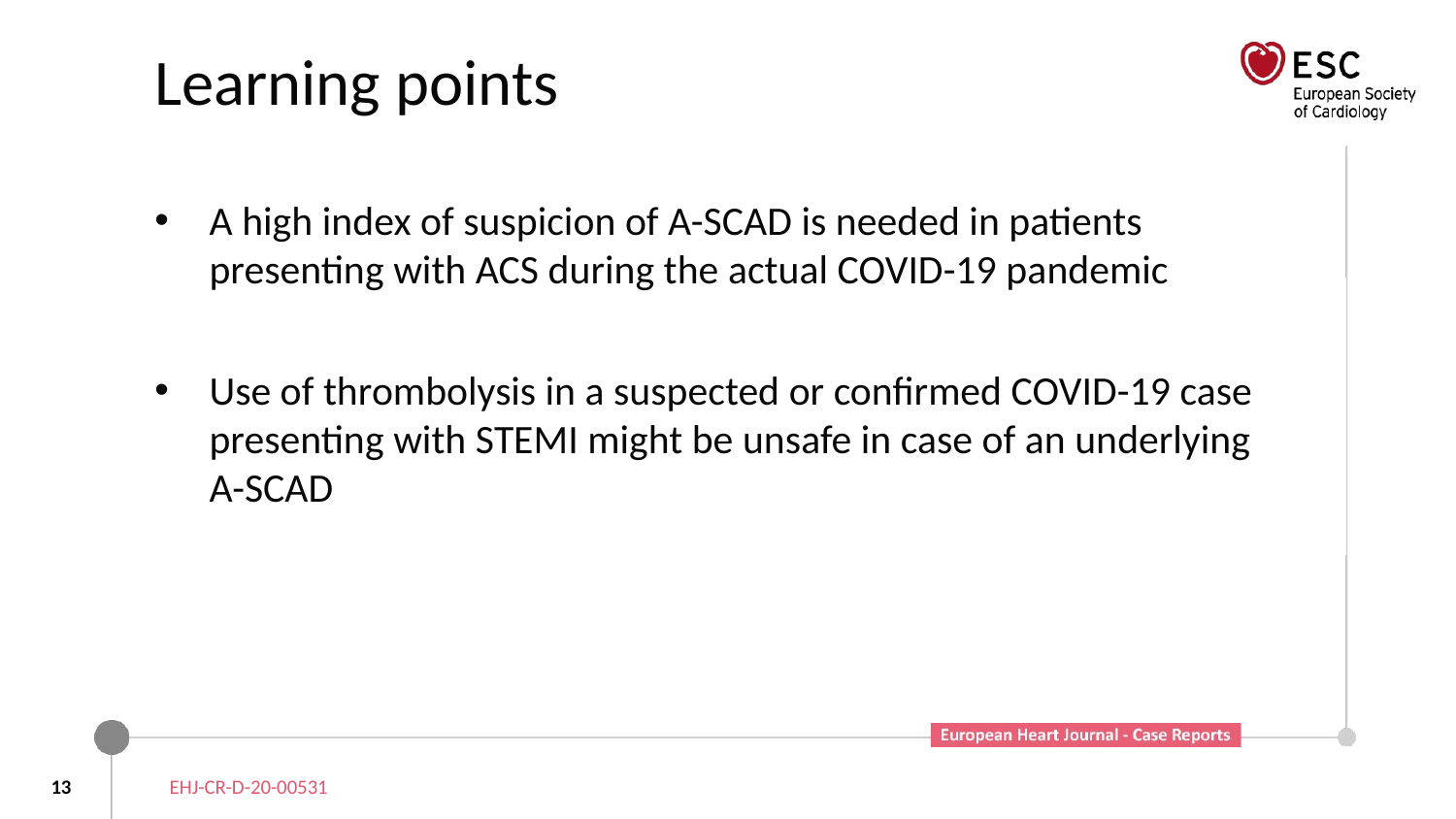

# Learning points
A high index of suspicion of A-SCAD is needed in patients presenting with ACS during the actual COVID-19 pandemic
Use of thrombolysis in a suspected or confirmed COVID-19 case presenting with STEMI might be unsafe in case of an underlying A-SCAD
13
EHJ-CR-D-20-00531

## Slide 14
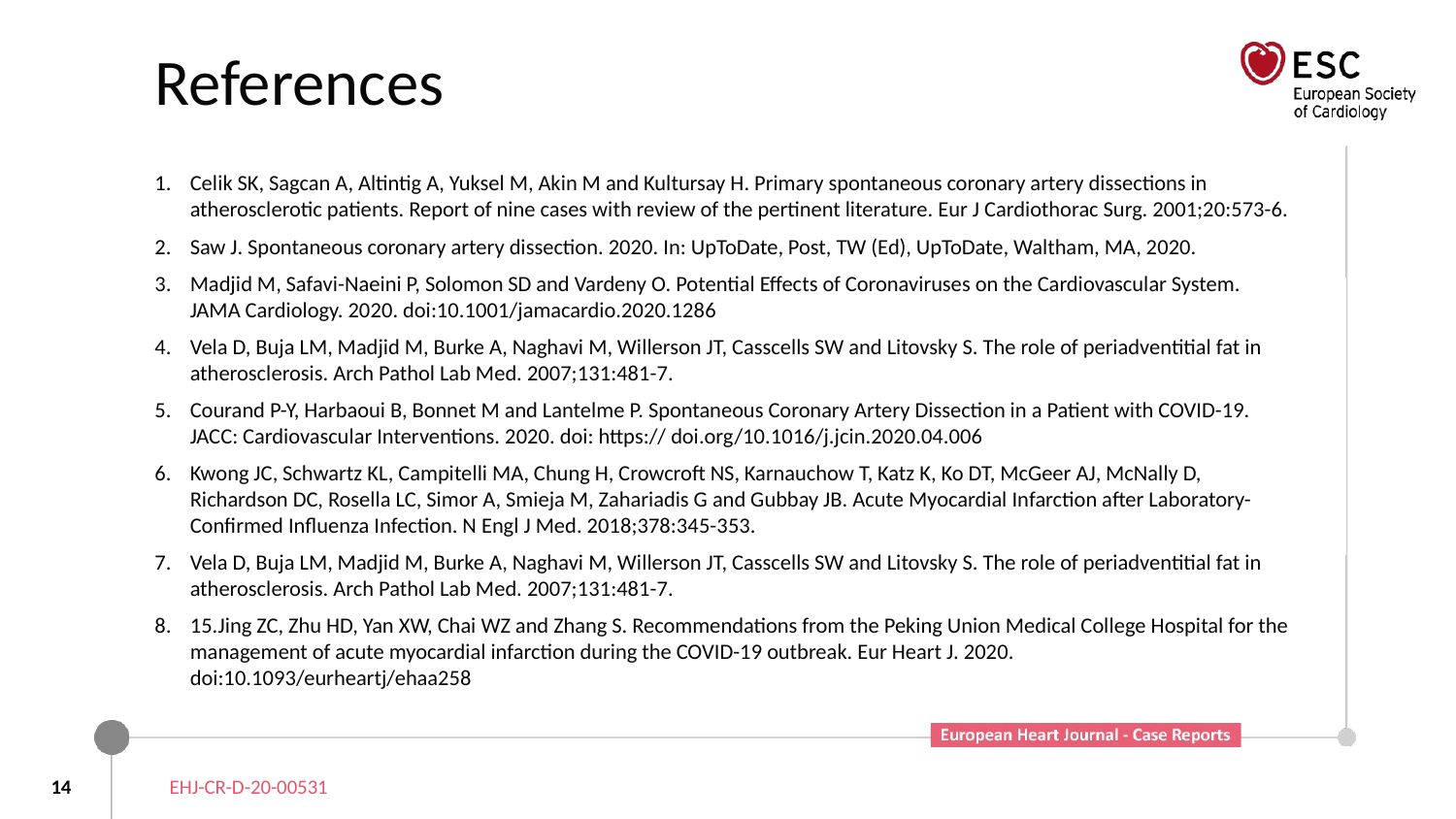

# References
Celik SK, Sagcan A, Altintig A, Yuksel M, Akin M and Kultursay H. Primary spontaneous coronary artery dissections in atherosclerotic patients. Report of nine cases with review of the pertinent literature. Eur J Cardiothorac Surg. 2001;20:573-6.
Saw J. Spontaneous coronary artery dissection. 2020. In: UpToDate, Post, TW (Ed), UpToDate, Waltham, MA, 2020.
Madjid M, Safavi-Naeini P, Solomon SD and Vardeny O. Potential Effects of Coronaviruses on the Cardiovascular System. JAMA Cardiology. 2020. doi:10.1001/jamacardio.2020.1286
Vela D, Buja LM, Madjid M, Burke A, Naghavi M, Willerson JT, Casscells SW and Litovsky S. The role of periadventitial fat in atherosclerosis. Arch Pathol Lab Med. 2007;131:481-7.
Courand P-Y, Harbaoui B, Bonnet M and Lantelme P. Spontaneous Coronary Artery Dissection in a Patient with COVID-19. JACC: Cardiovascular Interventions. 2020. doi: https:// doi.org/10.1016/j.jcin.2020.04.006
Kwong JC, Schwartz KL, Campitelli MA, Chung H, Crowcroft NS, Karnauchow T, Katz K, Ko DT, McGeer AJ, McNally D, Richardson DC, Rosella LC, Simor A, Smieja M, Zahariadis G and Gubbay JB. Acute Myocardial Infarction after Laboratory-Confirmed Influenza Infection. N Engl J Med. 2018;378:345-353.
Vela D, Buja LM, Madjid M, Burke A, Naghavi M, Willerson JT, Casscells SW and Litovsky S. The role of periadventitial fat in atherosclerosis. Arch Pathol Lab Med. 2007;131:481-7.
15.Jing ZC, Zhu HD, Yan XW, Chai WZ and Zhang S. Recommendations from the Peking Union Medical College Hospital for the management of acute myocardial infarction during the COVID-19 outbreak. Eur Heart J. 2020. doi:10.1093/eurheartj/ehaa258
14
EHJ-CR-D-20-00531
